# The relationship between serum ferritin level and clinical outcomes in sepsis based on a large public database

**DOI:** 10.1038/s41598-023-35874-2

**Published:** 2023-05-29

**Authors:** Liudang He, Cuirong Guo, Yingjie Su, Ning Ding

**Affiliations:** Department of Emergency Medicine, The Affiliated Changsha Central Hospital, Hengyang Medical School, University of South China, No.161 Shaoshan South Road, Changsha, 410004 Hunan China

**Keywords:** Risk factors, Biomarkers, Epidemiology, Outcomes research, Infectious-disease diagnostics

## Abstract

This study aimed to investigate the relationship between serum ferritin level and prognosis in sepsis. It also explored the potential prognostic value of serum ferritin for predicting outcomes in sepsis based on a large public database. Sepsis patients in MIMIC-IV database were included. Different models including crude model (adjusted for none), model I (adjusted for age and gender) and model II (adjusted for all potential confounders) were performed. Smooth fitting curves were constructed for exploring the relationships between serum ferritin and mortalities of 28-day, 90-day, 180-day and 1-year. Receiver operator characteristic (ROC) curve analysis was utilized for assessing the predictive value of serum ferritin. 1947 sepsis patients were included. The mortalities of 28-day, 90-day, 180-day and 1-year were 20.18% (n = 393), 28.35% (n = 552), 30.30% (n = 590) and 31.54% (n = 614), respectively. In Model II (adjusted for all potential confounders), for every 1000 ng/ml increment in serum ferritin, the values of OR in mortalities of in 28-day, 90-day, 180-day and 1-year were 1.13 (95% CI 1.07–1.19, *P* < 0.0001), 1.15 (95% CI 1.09–1.21, *P* < 0.0001), 1.16 (95% CI 1.10–1.22, *P* < 0.0001) and 1.17 (95% CI 1.10–1.23, *P* < 0.0001), respectively. The relationships between serum ferritin level and outcomes were non-linear. The areas under the ROC curve (AUC) of ferritin for predicting mortalities of 28-day, 90-day, 180-day and 1-year were 0.597 (95% CI 0.563–0.629), 0.593 (95% CI 0.564–0.621), 0.595 (95% CI 0.567–0.623) and 0.592 (95% CI 0.564–0.620), respectively. The non-linear relationships between serum ferritin and clinical outcomes in sepsis were found. Serum ferritin had a predictive value for short-term and long-term outcomes in sepsis.

## Introduction

Ferritin, as an iron storge protein, is broadly distributed in the cells, organs and circulation^1^. It has a significant role in maintaining iron homeostasis and helps to transport oxygen, produce energy and red blood cells^2^. In serum, ferritin level is usually regulated by various factors including iron imbalance, hormones and inflammatory cytokines^3^. Researches showed that serum ferritin not only could be an indicator for differentiating anemia^4^, but also was associated with numerous other diseases including malignant tumor^5^, metabolic syndrome^6^, digestive disease^7^ and even COVID-19^8^.

Ferritin, as an indispensable factor of the immune system, is capable of indicating the cellular defensive response to inflammation^9^. Increased ferritin was identified as both an acute phase reactant and a mediator of immune dysregulation due to infection^10^. Serum ferritin was a marker of cellular injury and related with prognosis in infectious diseases^11,12^. In critically ill patients, elevated levels of serum ferritin were associated with risk factors of worse outcomes including thrombocytopenia, hypoalbuminemia and life support managements^13^. Sepsis, as dysregulated organ dysfunction due to infection, usually stimulates high levels of inflammatory cytokines, which might raise serum ferritin^14^.

Since ferritin was closely related to infection, we aimed to investigate the relationship between serum ferritin level and prognosis and explored the prognostic value of serum ferritin for predicting short-term and long-term outcomes in sepsis based on a large public database.

## Methods

### Database

This retrospective study was performed on the basis of the data from the Medical Information Mart for Intensive Care IV(MIMIC-IV) database (https://mimic.mit.edu/iv/). MIMIC-IV is a U.S. public database and contains the clinical information of critical ill patients admitted in intensive care unit (ICU) of Beth Israel Deaconess Medical Center of Boston between 2008 and 2019^15–17^. The corresponding author (N.D.) passed the Protecting Human Research Participants exam (No.32900964) and had the permission for utilizing the MIMIC-IV database.

### Study design

We enrolled the septic patients from MIMIC-IV for data analysis. The definition of Sepsis 3.0 was applied for confirming the sepsis, which indicated that sepsis was diagnosed with infection and sequential organ failure assessment (SOFA) score ≥ 2 points^18^. Patients who met the criteria as follow were excluded: (1) missing data of serum ferritin within 24 h after admission; (2) missing data > 5% individual variables; (3) < 18 years old.

### Information and variables

Variables were extracted within 24 h after admission and only the first record of each variable was utilized.

The following information and variables were extracted: age, gender, marital status, ethnicity, comorbidities, organ dysfunction (acute kidney injury (AKI), septic shock), managements (the utility of vasopressors and ventilator, renal replacement therapy (RRT)), scores of SOFA and chronic health evaluation (APACHEII), length of stay (LOS) in ICU and hospital, prognosis (28-day mortality, 90-day mortality, 180-day mortality and 1-year mortality), vital signs(systolic blood pressure (SBP), diastolic blood pressure (DBP), heart rate (HR), respiratory rate (RR)), ferritin, total calcium, alanine aminotransferase (ALT), aspartate aminotransferase (AST), prothrombin time (PT), thrombin time (TT), creatinine, urea nitrogen, red blood cell (RBC), white blood cell (WBC), hemoglobin, hematocrit, red blood cell distribution width (RDW), platelet (PLT), anion gap (AG), bicarbonate, chloride and sodium.

### Statistical analysis

EmpowerStats (http://www.empowerstats.com) and the software packages R (http://www.R-project.org) were applied for statistical analysis. Statistically significant was considered when the P value was less than 0.05.

The septic patients were divided into four group (Q1 (≤ 244 ng/ml, n = 486), Q2 (245–542 ng/ml, n = 485), Q3 (543–1124 ng/ml, n = 488), Q4 (≥ 1125 ng/ml, n = 488)) based on the quartiles of serum ferritin level (Table 1). Different variables were expressed as follow: medians for continuous variables, and percentages or frequencies for categories variables. Chi-squared test and Mann–Whitney U-test were applied for variables analysis between four groups. Univariate analysis for clinical outcomes including 28-day mortality, 90-day mortality, 180-day mortality and 1-year mortality was performed. Associations between serum ferritin and outcomes were investigated in three models: crude model (adjusted for none), model I (adjusted for age and gender) and model II (adjusted for all potential confounders). Covariates were included as potential confounders in the final models if they changed the estimates of ferritin on 1-year mortality in sepsis by more than 10% or were significantly associated with clinical outcomes in sepsis^19,20^. The calculating steps were showed in Supplementary materials. The following covariates were selected a priori on the basis of established associations and/or plausible biological relations and tested: age; ALT; AG; total calcium; creatinine; hematocrit; hemoglobin; PLT; PT; TT; RDW; RBC; urea nitrogen; renal disease; APAHCEII; SOFA. In addition, gender, as a common confounder in many previous studies^21,22^, was also added to be adjusted in the final models.

**Table 1:** Comparison of different variables between the groups.

Ferritin (ng/ml) (quartiles)						
Variables	Total	Q1 (≤ 244)	Q2 (245–542)	Q3 (543–1124)	Q4 (≥ 1125)	*P*-value
Number	1947	486	485	488	488	
Age (years) (median, IQR)	64.00 (53.00–74.00)	65.00 (55.00–77.00)	65.00 (54.00–76.00)	65.00 (54.00–73.25)	62.00 (51.00–70.00)	< 0.001
Gender (n, %)						< 0.001
Female	867 (44.53%)	265 (54.53%)	234 (48.25%)	180 (36.89%)	188 (38.52%)	
Male	1080 (55.47%)	221 (45.47%)	251 (51.75%)	308 (63.11%)	300 (61.48%)	
Comorbidities (n, %)						
Hypertension	341 (17.51%)	71 (14.61%)	100 (20.62%)	82 (16.80%)	88 (18.03%)	0.096
CAD	160 (8.22%)	47 (9.67%)	42 (8.66%)	42 (8.61%)	29 (5.94%)	0.177
Renal disease	94 (4.83%)	7 (1.44%)	21 (4.33%)	34 (6.97%)	32 (6.56%)	< 0.001
Diabetes	63 (3.24%)	17 (3.50%)	11 (2.27%)	26 (5.33%)	9 (1.84%)	0.010
Vital signs (median, IQR)						
SBP (mmHg)	111.00 (99.00–129.00)	110.00 (97.00–128.00)	112.00 (99.00–130.00)	112.00 (98.00–128.00)	112.50 (99.00–130.00)	0.429
DBP (mmHg)	63.00 (53.00–74.00)	62.00 (52.00–73.00)	63.00 (55.00–73.00)	62.00 (53.00–74.00)	65.00 (54.00–77.00)	0.147
HR (beats/min)	97.00 (83.00–113.00)	95.00 (81.25–111.00)	98.00 (80.00–112.00)	97.00 (82.00–113.00)	100.00 (86.00–117.00)	0.014
RR (beats/min)	21.00 (17.00–25.00)	21.00 (17.00–25.00)	21.00 (17.00–26.00)	21.00 (17.00–26.00)	21.00 (18.00–25.00)	0.447
Laboratory findings (median, IQR)						
Ferritin (ng/ml)	542.00 (244.00–1125.00)	138.50 (86.00–192.75)	375.00 (308.00–445.00)	782.00 (643.00–931.50)	1920.50 (1452.75–3359.50)	< 0.001
Total calcium (mg/dl)	8.00 (7.50–8.60)	8.10 (7.60–8.70)	8.00 (7.50–8.60)	8.00 (7.40–8.60)	8.00 (7.40–8.60)	0.059
ALT (IU/l)	28.00 (16.00–62.00)	24.00 (15.00–48.00)	25.00 (15.00–47.00)	29.00 (16.00–61.00)	42.00 (20.00–106.00)	< 0.001
AST (IU/l)	44.00 (24.00–93.00)	35.00 (21.00–69.00)	38.00 (23.00–69.00)	44.50 (24.75–97.50)	63.50 (30.00–160.50)	< 0.001
PT (s)	15.40 (13.30–20.10)	15.60 (13.10–19.40)	15.10 (13.20–19.40)	15.30 (13.40–19.70)	15.60 (13.50–21.95)	0.079
TT(s)	32.80 (28.60–40.80)	32.90 (28.70–39.95)	33.20 (28.90–42.20)	32.40 (28.20–40.40)	32.90 (28.67–42.65)	0.358
Creatinine (mg/dl)	1.40 (0.90–2.50)	1.30 (0.80–2.00)	1.40 (0.90–2.40)	1.60 (0.90–2.90)	1.50 (0.90–3.12)	< 0.001
Urea nitrogen (mg/dl)	29.00 (18.00–50.00)	28.00 (17.00–48.00)	28.00 (18.00–47.00)	31.00 (18.00–54.00)	29.00 (18.00–51.00)	0.207
RBC (*10^12^/l)	3.31 (2.85–3.88)	3.43 (2.94–3.95)	3.40 (2.89–3.99)	3.33 (2.85–3.84)	3.16 (2.71–3.68)	< 0.001
WBC (*10^9^/l)	11.50 (7.40–17.15)	11.00 (7.40–16.00)	11.90 (8.00–17.40)	12.40 (8.00–18.20)	10.70 (6.60–16.97)	0.033
Hemoglobin (g/dl)	9.80 (8.40–11.30)	9.60 (8.20–10.90)	10.00 (8.60–11.60)	10.10 (8.70–11.50)	9.60 (8.20–11.30)	< 0.001
Hematocrit (%)	30.20 (26.10–34.65)	30.15 (26.05–34.40)	30.50 (26.50–35.40)	30.50 (26.60–34.80)	29.30 (25.30–34.20)	0.011
RDW (%)	15.70 (14.30–17.70)	16.20 (14.90–18.20)	15.40 (14.00–17.30)	15.50 (14.10–17.30)	15.70 (14.20–17.90)	< 0.001
PLT (*10^9^/l)	184.00 (117.00–275.00)	204.50 (143.50–293.75)	193.00 (125.00–292.00)	180.00 (112.75–265.50)	151.00 (93.75–248.00)	< 0.001
AG (mmol/l)	16.00 (13.00–19.00)	15.00 (13.00–18.00)	16.00 (13.00–18.00)	16.00 (13.00–19.00)	16.00 (13.75–20.00)	0.012
Bicarbonate (mmol/l)	21.00 (18.00–24.00)	22.00 (18.00–25.00)	21.00 (18.00–25.00)	21.00 (18.00–24.00)	21.00 (18.00–24.00)	0.152
Chloride (mmol/l)	102.00 (98.00–107.00)	103.00 (98.00–108.00)	103.00 (99.00–108.00)	103.00 (98.00–108.00)	101.00 (96.00–106.00)	< 0.001
Sodium (mmol/l)	137.00 (134.00–141.00)	138.00 (134.25–141.00)	138.00 (134.00–141.00)	138.00 (134.00–141.00)	136.00 (133.00–140.00)	< 0.001
Scoring systems (median, IQR)						
APACHEII	12.00 (9.00–15.00)	12.00 (9.00–15.00)	12.00 (9.00–15.00)	13.00 (10.00–15.00)	12.00 (9.00–15.00)	0.011
SOFA	3.00 (2.00–5.00)	2.00 (2.00–4.00)	3.00 (2.00–4.00)	3.00 (2.00–5.00)	4.00 (2.00–6.00)	< 0.001
Organ dysfunction (n, %)						
Septic shock	845(43.40%)	236 (48.56%)	189 (38.97%)	201 (41.19%)	219 (44.88%)	0.014
AKI	1475(75.76%)	341 (70.16%)	363 (74.85%)	384 (78.69%)	387 (79.30%)	0.003
Clinical outcomes (days) (median, IQR)						
LOS in ICU	6.72 (2.85–14.63)	4.61 (2.46–10.84)	6.04 (2.75–12.84)	8.64 (3.67–16.79)	8.11 (3.07–17.10)	< 0.001
LOS in hospital	17.28 (9.45–29.29)	14.86 (8.48–26.87)	16.92 (8.96–27.40)	18.99 (10.81–30.90)	17.82 (9.89–33.54)	< 0.001
28-day mortality (n, %)	393 (20.18%)	77 (15.84%)	73 (15.05%)	99 (20.29%)	144 (29.51%)	< 0.001
90-day mortality (n, %)	552 (28.35%)	109 (22.43%)	112 (23.09%)	140 (28.69%)	191 (39.14%)	< 0.001
180-day mortality (n, %)	590 (30.30%)	117 (24.07%)	118 (24.33%)	153 (31.35%)	202 (41.39%)	< 0.001
1-year mortality (n, %)	614 (31.54%)	123 (25.31%)	125 (25.77%)	159 (32.58%)	207 (42.42%)	< 0.001

ALT, alanine aminotransferase; AST, aspartate aminotransferase; CAD, coronary artery disease; SBP, systolic blood pressure; DBP, diastolic blood pressure; HR, heart rate; RR, respiratory rate; WBC, white blood cells; PLT, platelet, RDW, red blood cell distribution width, RBC, red blood cells; PT, prothrombin time; TT, thrombin time; AG, anion gap; SOFA, sequential organ failure assessment; APACHE, acute physiology and chronic health evaluation; AKI, acute kidney injury; LOS, length of stay; ICU, intensive care unit; IQR, interquartile ranges.

In addition, two models including model A (linear model) and model B (two-segment nonlinear model) were utilized for comparison and the better one was selected based on the *P* value. If < 0.05, the nonlinear model was the better and the turning point of serum ferritin was calculated. The smooth fitting curves were performed for indicating the relationships between serum ferritin level and outcomes. Kaplan–Meier analysis for survival probability in four groups (Q1–Q4) was constructed. Subgroup analysis was done for investigating the stability of the results. The receiver-operator characteristic (ROC) analysis of serum ferritin for predicting outcomes were performed. The different performances of ferritin, SOFA score and APAHCEII score including specificity, sensitivity and cut-off value were analyzed.

### Ethical approval

This study was conducted in accordance with Declaration of Helsinki 2002. MIMIC-IV was an anonymized public database. To apply for access to the database, we passed the Protecting Human Research Participants exam (No.32900964). The project was approved by the institutional review boards of the Massachusetts Institute of Technology (MIT) and Beth Israel Deaconess Medical Center (BIDMC) and was given a waiver of informed consent.

## Results

### Description of the sepsis cohort and comparison of variables

1947 sepsis patients were included in our study (Supplementary Fig. 1 and Supplementary Table 1). The median age was 64 years old and the proportion of males was 55.47% (n = 867). 40.16% of the patients were married and 65.07% were White. The in-hospital incidences of AKI and septic shock were 75.76% and 43.40%, respectively. Of the sepsis patients, 22.85% had RRT use, 26.50% had vasopressors use and 83.82% had ventilation supply. The mortalities of 28-day, 90-day, 180-day and 1-year were 20.18% (n = 393), 28.35% (n = 552), 30.30% (n = 590) and 31.54% (n = 614), respectively.

In Table 1, we compared the variables between four groups based on the quartiles of serum ferritin level. Significant differences were showed in age (*P* < 0.001), gender (*P* < 0.001), renal disease (*P* < 0.001), diabetes (*P* = 0.010), HR (*P* = 0.014), ALT (*P* < 0.001), AST (*P* < 0.001), creatinine (*P* < 0.001), RBC (*P* < 0.001), WBC (*P* = 0.033), hemoglobin (*P* < 0.001), hematocrit (*P* = 0.011), RDW (*P* < 0.001), PLT (*P* < 0.001), AG (*P* = 0.012), chloride (*P* < 0.001), sodium (*P* < 0.001), APCHEII score (*P* = 0.011) and SOFA score (*P* < 0.001). In Q4 group, the levels of ALT, AST and SOFA score were significantly higher, while the levels of RBC and PLT were significantly lower compared to other three (Q1-Q3) groups. The incidences of septic shock in Q1–Q4 groups were 48.56%, 38.97%, 41.19% and 44.88%, respectively (*P* = 0.014). The incidences of AKI in Q1-Q4 groups were 70.16%, 74.85%, 78.69% and 79.30%, respectively (*P* = 0.003).

The median days of LOS in ICU and hospital were 6.72 and 17.28, respectively. The mortalities of in 28-day, 90-day, 180-day and 1-year Q4 groups were the highest compared to Q1-Q3 groups, which were 29.51%, 39.14%, 41.39% and 42.42%, respectively.

### Univariate analyses for different outcomes in sepsis

In Table 2, univariate analyses were implemented for different outcomes. Variables were significantly associated with all four different outcomes in sepsis (all P < 0.05) as follow: age, total calcium, PT, TT, urea nitrogen, RBC, hemoglobin, RDW, PLT, AG, chloride, APACHEII score and SOFA score. For ferritin (per 1000 ng/ml increase), the values of OR in mortalities of in 28-day, 90-day, 180-day and 1-year were 1.13 (95% CI 1.08–1.18, *P* < 0.0001), 1.14 (95% CI 1.09–1.19, *P* < 0.0001), 1.15 (95% CI 1.10–1.20, *P* < 0.0001) and 1.15 (95% CI 1.10–1.21, *P* < 0.0001), respectively.

**Table 2:** Univariate analysis for different outcomes in sepsis patients.

Variables	Univariable (OR 95% CI *P*) (28-day mortality)	Univariable (OR 95% CI *P*) (90-day mortality)	Univariable (OR 95% CI *P*) (180-day mortality)	Univariable (OR 95% CI *P*) (1-year mortality)
Age(years)	1.01 (1.01, 1.02) 0.0005	1.01 (1.00, 1.02) 0.0085	1.01 (1.00, 1.02) 0.0053	1.01 (1.00, 1.01) 0.0119
Gender				
Male	Ref	Ref	Ref	Ref
Female	0.93 (0.74, 1.16) 0.4953	1.07 (0.87, 1.30) 0.5309	1.02 (0.84, 1.24) 0.8216	1.00 (0.82, 1.21) 0.9676
Hypertension				
No	Ref	Ref	Ref	Ref
Yes	0.86 (0.63, 1.16) 0.3106	0.87 (0.67, 1.14) 0.3100	0.90 (0.69, 1.16) 0.4114	0.91 (0.71, 1.18) 0.4775
CAD				
No	Ref	Ref	Ref	Ref
Yes	1.03 (0.69, 1.54) 0.8849	1.16 (0.82, 1.65) 0.3962	1.12 (0.79, 1.58) 0.5281	1.19 (0.84, 1.67) 0.3254
Renal disease				
No	Ref	Ref	Ref	Ref
Yes	1.15 (0.70, 1.89) 0.5938	1.20 (0.77, 1.87) 0.4325	1.46 (0.95, 2.23) 0.0854	1.44 (0.94, 2.20) 0.0957
Diabetes				
No	Ref	Ref	Ref	Ref
Yes	0.83 (0.43, 1.61) 0.5844	0.78 (0.44, 1.41) 0.4172	0.92 (0.53, 1.60) 0.7612	0.94 (0.54, 1.62) 0.8110
SBP (mmHg)	1.00 (0.99, 1.00) 0.2932	1.00 (0.99, 1.00) 0.3220	1.00 (0.99, 1.00) 0.3904	1.00 (0.99, 1.00) 0.5125
DBP (mmHg)	1.00 (0.99, 1.00) 0.2173	1.00 (0.99, 1.00) 0.7550	1.00 (0.99, 1.00) 0.7568	1.00 (0.99, 1.01) 0.9743
HR (beats/min)	1.00 (0.99, 1.00) 0.7442	1.00 (1.00, 1.01) 0.3082	1.00 (1.00, 1.01) 0.4467	1.00 (1.00, 1.01) 0.4307
RR (beats/min)	1.01 (0.99, 1.02) 0.3977	1.00 (0.99, 1.02) 0.6243	1.00 (0.99, 1.02) 0.5885	1.01 (0.99, 1.02) 0.4668
Ferritin (per 1000 ng/ml increase)	1.13 (1.08, 1.18) < 0.0001	1.14 (1.09, 1.19) < 0.0001	1.15 (1.10, 1.20) < 0.0001	1.15 (1.10, 1.21) < 0.0001
Total calcium (mg/dl)	1.15 (1.03, 1.29) 0.0135	1.21 (1.10, 1.34) 0.0001	1.21 (1.10, 1.34) 0.0002	1.22 (1.10, 1.34) < 0.0001
ALT (IU/l)	1.00 (1.00, 1.00) 0.4292	1.00 (1.00, 1.00) 0.2807	1.00 (1.00, 1.00) 0.3927	1.00 (1.00, 1.00) 0.5037
AST (IU/l)	1.00 (1.00, 1.00) 0.0431	1.00 (1.00, 1.00) 0.0574	1.00 (1.00, 1.00) 0.0890	1.00 (1.00, 1.00) 0.1323
PT (s)	1.01 (1.00, 1.02) 0.0027	1.01 (1.00, 1.02) 0.0158	1.01 (1.00, 1.02) 0.0169	1.01 (1.00, 1.02) 0.0115
TT (s)	1.01 (1.01, 1.02) < 0.0001	1.01 (1.01, 1.02) < 0.0001	1.01 (1.01, 1.02) < 0.0001	1.01 (1.01, 1.02) < 0.0001
Creatinine (mg/dl)	1.04 (0.99, 1.10) 0.1439	1.01 (0.96, 1.06) 0.8304	1.02 (0.97, 1.07) 0.5437	1.02 (0.97, 1.07) 0.3937
Urea nitrogen (mg/dl)	1.01 (1.00, 1.01) < 0.0001	1.01 (1.00, 1.01) 0.0001	1.01 (1.00, 1.01) 0.0001	1.01 (1.00, 1.01) < 0.0001
RBC (*10^12^/l)	0.75 (0.65, 0.88) 0.0003	0.78 (0.69, 0.90) 0.0003	0.75 (0.66, 0.86) < 0.0001	0.75 (0.66, 0.85) < 0.0001
WBC (*10^9^/l)	1.01 (0.99, 1.02) 0.2875	1.00 (0.99, 1.01) 0.9770	1.00 (0.99, 1.01) 0.5329	0.99 (0.98, 1.00) 0.2260
Hemoglobin (g/dl)	0.94 (0.90, 1.00) 0.0340	0.95 (0.91, 1.00) 0.0356	0.94 (0.89, 0.98) 0.0053	0.94 (0.89, 0.98) 0.0050
Hematocrit (%)	0.98 (0.97, 1.00) 0.0531	0.99 (0.97, 1.00) 0.0727	0.98 (0.97, 1.00) 0.0175	0.98 (0.97, 1.00) 0.0150
RDW (%)	1.16 (1.12, 1.20) < 0.0001	1.17 (1.13, 1.22) < 0.0001	1.18 (1.14, 1.22) < 0.0001	1.17 (1.13, 1.22) < 0.0001
PLT (*10^9^/l)	1.00 (1.00, 1.00) 0.0003	1.00 (1.00, 1.00) 0.0002	1.00 (1.00, 1.00) 0.0003	1.00 (1.00, 1.00) < 0.0001
AG (mmol/l)	1.04 (1.02, 1.07) < 0.0001	1.03 (1.01, 1.05) 0.0012	1.03 (1.01, 1.05) 0.0050	1.02 (1.01, 1.04) 0.0094
Bicarbonate (mmol/l)	1.01 (0.99, 1.03) 0.4945	1.02 (1.00, 1.04) 0.0711	1.02 (1.00, 1.04) 0.0796	1.02 (1.00, 1.03) 0.1076
Chloride (mmol/l)	0.97 (0.95, 0.98) < 0.0001	0.97 (0.96, 0.99) 0.0001	0.98 (0.96, 0.99) 0.0004	0.98 (0.97, 0.99) 0.0015
Sodium (mmol/l)	0.99 (0.97, 1.01) 0.2134	0.99 (0.98, 1.01) 0.4187	0.99 (0.98, 1.01) 0.3703	0.99 (0.98, 1.01) 0.4520
APACHEII	1.06 (1.03, 1.09) < 0.0001	1.04 (1.02, 1.07) 0.0002	1.05 (1.02, 1.07) < 0.0001	1.05 (1.03, 1.07) < 0.0001
SOFA	1.22 (1.16, 1.28) < 0.0001	1.19 (1.14, 1.25) < 0.0001	1.18 (1.13, 1.24) < 0.0001	1.19 (1.13, 1.24) < 0.0001

ALT, alanine aminotransferase; AST, aspartate aminotransferase; CAD, coronary artery disease; SBP, systolic blood pressure; DBP, diastolic blood pressure; HR, heart rate; RR, respiratory rate; WBC, white blood cells; PLT, platelet; RDW, red blood cell distribution width; RBC, red blood cells; PT, prothrombin time; TT, thrombin time; AG, anion gap; APACHE, acute physiology and chronic health evaluation; SOFA, sequential organ failure assessment; OR, odds ratio; CI, confidential interval.

### Association between serum ferritin and outcomes in different models

Table 3 demonstrated the association between serum ferritin and outcomes including mortalities of in 28-day, 90-day, 180-day and 1-year in sepsis. In Model II (adjusted for all potential confounders), for every 1000 ng/ml increment in serum ferritin, the values of OR in mortalities of in 28-day, 90-day, 180-day and 1-year were 1.13 (95% CI 1.07–1.19, *P* < 0.0001), 1.15 (95% CI 1.09–1.21, *P* < 0.0001), 1.16 (95% CI 1.10–1.22, *P* < 0.0001) and 1.17 (95% CI 1.10–1.23, *P* < 0.0001), respectively. In addition, we changed the serum ferritin from continuous variable to categorial variable (Q1–Q4) and analyzed the associations in three models. Compared Q1 group, the risk of mortalities of 28-day, 90-day, 180-day and 1-year increased significantly in Q4 group in all three models (all values of *P* for trend < 0.0001). In model II, the values of OR in mortalities of in 28-day, 90-day, 180-day and 1-year were 2.16 (95% CI 1.53–3.07, *P* < 0.0001), 2.31 (95% CI 1.69–3.17, *P* < 0.0001), 2.30 (95% CI 1.69–3.14, *P* < 0.0001) and 2.19 (95% CI 1.61–2.97, *P* < 0.0001) in Q4 group, respectively.

**Table 3:** Associations between ferritin and different clinical outcomes in three models.

Exposure	Crude model	Model I	Model II
	OR (95% CI), *P*-value	OR(95% CI), *P*-value	OR (95% CI), *P*-value
28-day mortality			
Ferritin (per 1000 ng/ml increment)	1.13 (1.08, 1.18) < 0.0001	1.14 (1.09, 1.20) < 0.0001	1.13 (1.07, 1.19) < 0.0001
Ferritin (ng/ml)quartiles			
Q1	Ref	Ref	Ref
Q2	0.94 (0.66, 1.33) 0.7328	0.94 (0.67, 1.34) 0.7500	1.00 (0.69, 1.44) 0.9971
Q3	1.35 (0.97, 1.88) 0.0721	1.38 (0.99, 1.93) 0.0559	1.40 (0.98, 2.01) 0.0640
Q4	2.22 (1.63, 3.04) < 0.0001	2.40 (1.75, 3.30) < 0.0001	2.16 (1.53, 3.07) < 0.0001
P for trend	< 0.0001	< 0.0001	< 0.0001
90-day mortality			
Ferritin (per 1000 ng/ml increment)	1.14 (1.09, 1.19) < 0.0001	1.16 (1.11, 1.21) < 0.0001	1.15 (1.09, 1.21) < 0.0001
Ferritin (ng/ml)quartiles			
Q1	Ref	Ref	Ref
Q2	1.04 (0.77, 1.40) 0.8049	1.05 (0.78, 1.42) 0.7461	1.16 (0.84, 1.59) 0.3717
Q3	1.39 (1.04, 1.86) 0.0254	1.45 (1.08, 1.94) 0.0132	1.54 (1.12, 2.12) 0.0076
Q4	2.22 (1.68, 2.94) < 0.0001	2.40 (1.80, 3.19) < 0.0001	2.31 (1.69, 3.17) < 0.0001
P for trend	< 0.0001	< 0.0001	< 0.0001
180-day mortality			
Ferritin (per 1000 ng/ml increment)	1.15 (1.10, 1.20) < 0.0001	1.17 (1.11, 1.22) < 0.0001	1.16 (1.10, 1.22) < 0.0001
Ferritin (ng/ml)quartiles			
Q1	Ref	Ref	Ref
Q2	1.01 (0.76, 1.36) 0.9259	1.02 (0.76, 1.37) 0.8765	1.13 (0.83, 1.54) 0.4503
Q3	1.44 (1.09, 1.91) 0.0114	1.49 (1.12, 1.98) 0.0062	1.58 (1.16, 2.15) 0.0038
Q4	2.23 (1.69, 2.93) < 0.0001	2.39 (1.81, 3.16) < 0.0001	2.30 (1.69, 3.14) < 0.0001
P for trend	< 0.0001	< 0.0001	< 0.0001
1-year mortality			
Ferritin (per 1000 ng/ml increment)	1.15 (1.10, 1.21) < 0.0001	1.17 (1.11, 1.23) < 0.0001	1.17 (1.10, 1.23) < 0.0001
Ferritin (ng/ml)quartiles			
Q1	Ref	Ref	Ref
Q2	1.02 (0.77, 1.37) 0.8682	1.03 (0.77, 1.38) 0.8304	1.13 (0.83, 1.53) 0.4388
Q3	1.43 (1.08, 1.88) 0.0125	1.47 (1.11, 1.94) 0.0079	1.53 (1.13, 2.08) 0.0063
Q4	2.17 (1.66, 2.85) < 0.0001	2.31 (1.75, 3.04) < 0.0001	2.19 (1.61, 2.97) < 0.0001
*P* for trend	< 0.0001	< 0.0001	< 0.0001

Crude model adjusted for: None;

Model I adjusted for: age; gender;

Model II adjusted for: age; gender; ALT; AG; total calcium; creatinine; hematocrit; hemoglobin; PLT; PT; TT; RDW; RBC; urea nitrogen; renal disease; APAHCEII; SOFA.

ALT, alanine aminotransferase; PLT, platelet; RDW, red blood cell distribution width; RBC, red blood cells; PT, prothrombin time; TT, thrombin time; AG, anion gap; APACHE, acute physiology and chronic health evaluation; SOFA, sequential organ failure assessment; OR, odds ratio; CI, confidential interval.

### A non-linear relationship between serum ferritin and outcomes

**Figure 1:**
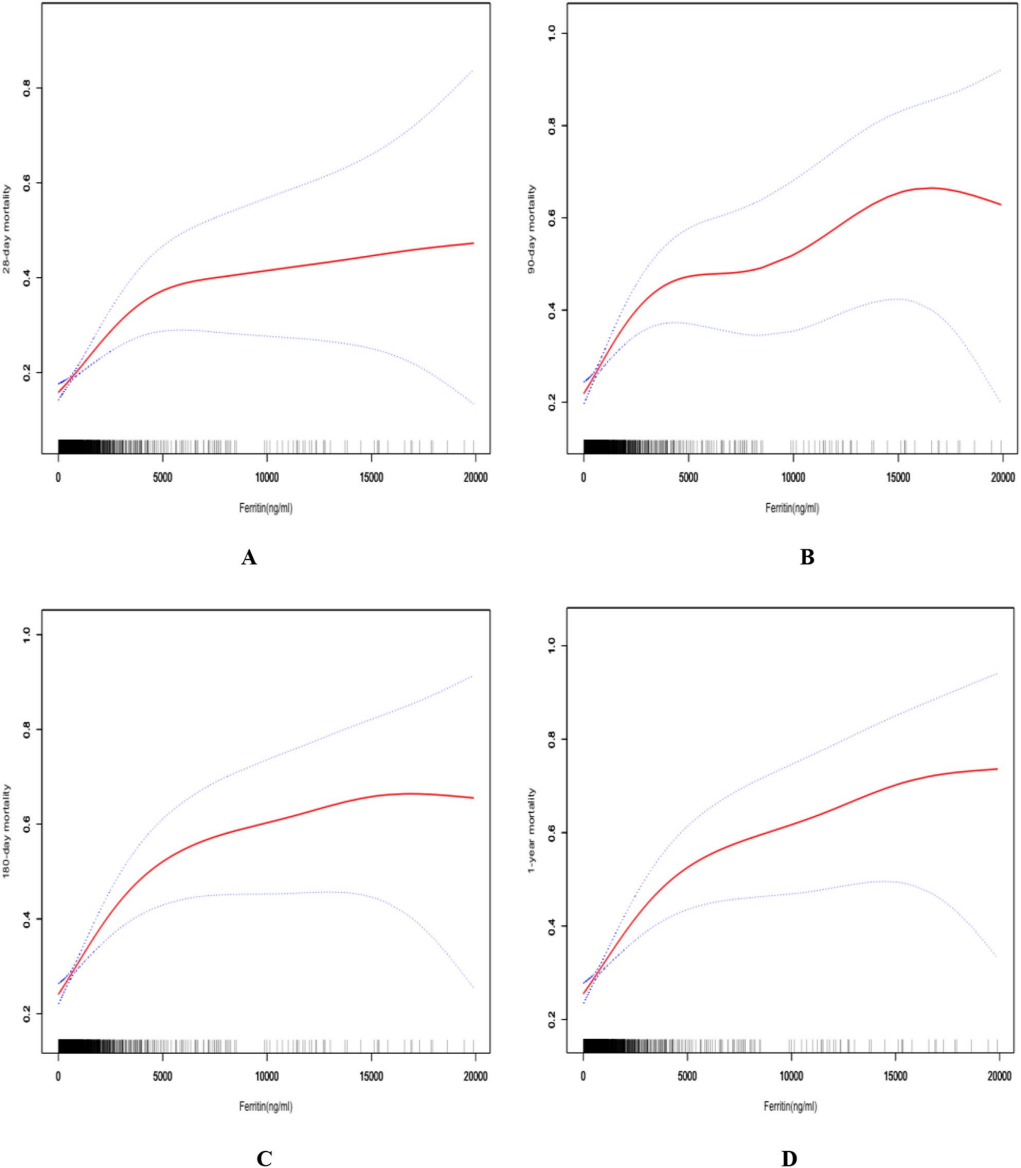
Smooth fitting curves demonstrated the non-linear relationships between serum ferritin level and mortalities of 28-day (**A**), 90-day (**B**), 180-day (**C**) and 1-year (**D**).

In Table 4, we compared two models including the linear model (model A) and two-segment non-linear model (model B) in all clinical outcomes and found that all the P values for the log-likelihood ratio test were less than 0.05, which indicated the non-linear model was better for expressing the association between serum ferritin and clinical outcomes. In Fig. 1, smooth fitting curves were constructed, which demonstrated the non-linear relationships between serum ferritin level and mortalities of 28-day (A), 90-day (B), 180-day (C) and 1-year (D). The turning points of serum ferritin in the four clinical outcomes were 2340 ng/ml, 2250 ng/ml, 2280 ng/ml and 2300 ng/ml, respectively.

**Table 4:** The threshold effect for analysis between ferritin and clinical outcomes.

	Number (%)	OR (95% CI), *P*-value
28-day mortality		
Model A: The linear model	1947 (100%)	1.13 (1.07, 1.19) < 0.0001
Model B: Two-segment non-linear model		
The turning point of ferritin (ng/ml)		
≤ 2340( slope 1)	1762 (90.50%)	1.54 (1.29, 1.85) < 0.0001
> 2340( slope 2)	185 (9.50%)	1.04 (0.97, 1.11) 0.2873
Slope 2 to slope 1		0.67 (0.54, 0.84) 0.0004
Predicted at 2340		− 0.68 (− 0.95, − 0.41)
*P* for the log-likelihood ratio test		< 0.001
90-day mortality		
Model A: The linear model	1947 (100%)	1.15 (1.09, 1.21) < 0.0001
Model B: Two-segment non-linear model		
The turning point of ferritin(ng/ml)		
≤ 2250( slope 1)	1754 (90.09%)	1.54 (1.30, 1.83) < 0.0001
> 2250( slope 2)	193 (9.91%)	1.06 (0.99, 1.13) 0.0967
Slope 2 to slope 1		0.69 (0.56, 0.84) 0.0004
Predicted at 2250		− 0.31 (− 0.56, − 0.06)
*P* for the log-likelihood ratio test		< 0.001
180-day mortality		
Model A: The linear model	1947 (100%)	1.16 (1.10, 1.22) < 0.0001
Model B: Two-segment non-linear model		
The turning point of ferritin(ng/ml)		
≤ 2280( slope 1)	1755 (90.14%)	1.56 (1.32, 1.84) < 0.0001
> 2280( slope 2)	192 (9.86%)	1.06 (1.00, 1.14) 0.0695
Slope 2 to slope 1		0.68 (0.56, 0.84) 0.0003
Predicted at		− 0.18 (− 0.43, 0.06)
*P* for the log-likelihood ratio test		< 0.001
1-year mortality		
Model A: The linear model	1947 (100%)	1.17 (1.10, 1.23) < 0.0001
Model B: Two-segment non-linear model		
The turning point of ferritin(ng/ml)		
≤ 2300(slope 1)	1756 (90.19%)	1.50 (1.27, 1.76) < 0.0001
> 2300(slope 2)	191 (9.81%)	1.08 (1.01, 1.16) 0.0281
Slope 2 to slope 1		0.72 (0.59, 0.88) 0.0017
Predicted at 2300		− 0.15 (− 0.40, 0.09)
*P* for the log-likelihood ratio test		0.002

Model A and B adjusted for: age; gender; ALT; AG; total calcium; creatinine; hematocrit; hemoglobin; PLT; PT; TT; RDW; RBC; urea nitrogen; renal disease; APAHCEII; SOFA.

ALT, alanine aminotransferase; PLT, platelet; RDW, red blood cell distribution width; RBC, red blood cells, PT, prothrombin time; TT, thrombin time; AG, anion gap; APACHE, acute physiology and chronic health evaluation; SOFA, sequential organ failure assessment; OR, odds ratio; CI, confidential interval.

### Kaplan–Meier analysis for survival probability

**Figure 2:**
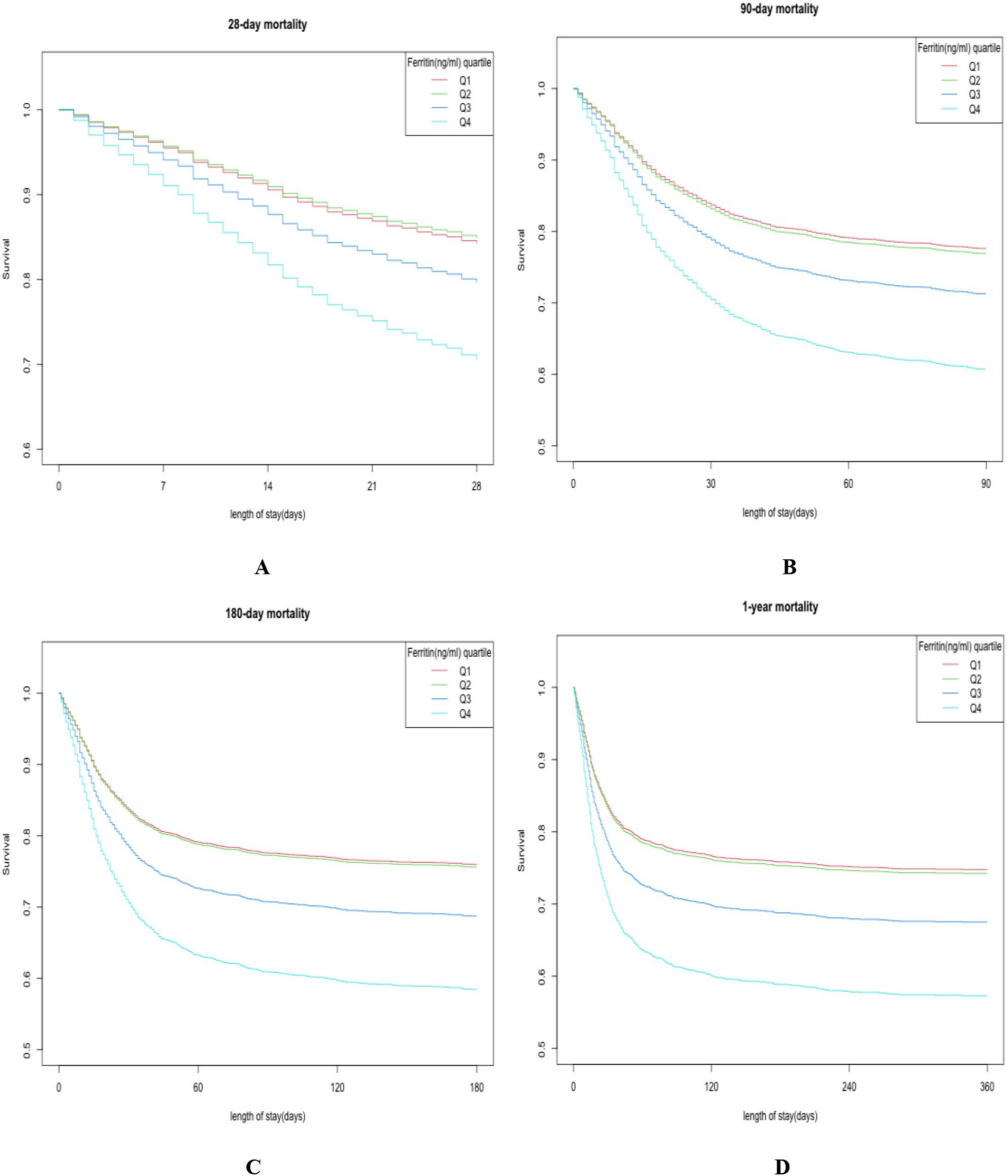
Kaplan–Meier analysis for cumulative hazard of mortalities of 28-day (**A**), 90-day (**B**), 180-day (**C**) and 1-year (**D**) in sepsis based on quartiles of serum ferritin level (Q1–Q4).

Figure 2 illuminated Kaplan–Meier analysis for survival probability in four groups (Q1–Q4). In Q4 group, the lowest survival probabilities in 28-day (A), 90-day (B), 180-day(C) and 1-year (D) were found (all *P* < 0.001).

### Subgroup analysis

In Supplementary Table 2, subgroup analysis was performed. The results were comparatively stable in most subgroups. Patients who were female with higher levels of WBC (≥ 15.1*10^9^/l) had higher risk of mortality.

### Prognostic value of ferritin in predicting clinical outcomes

**Figure 3:**
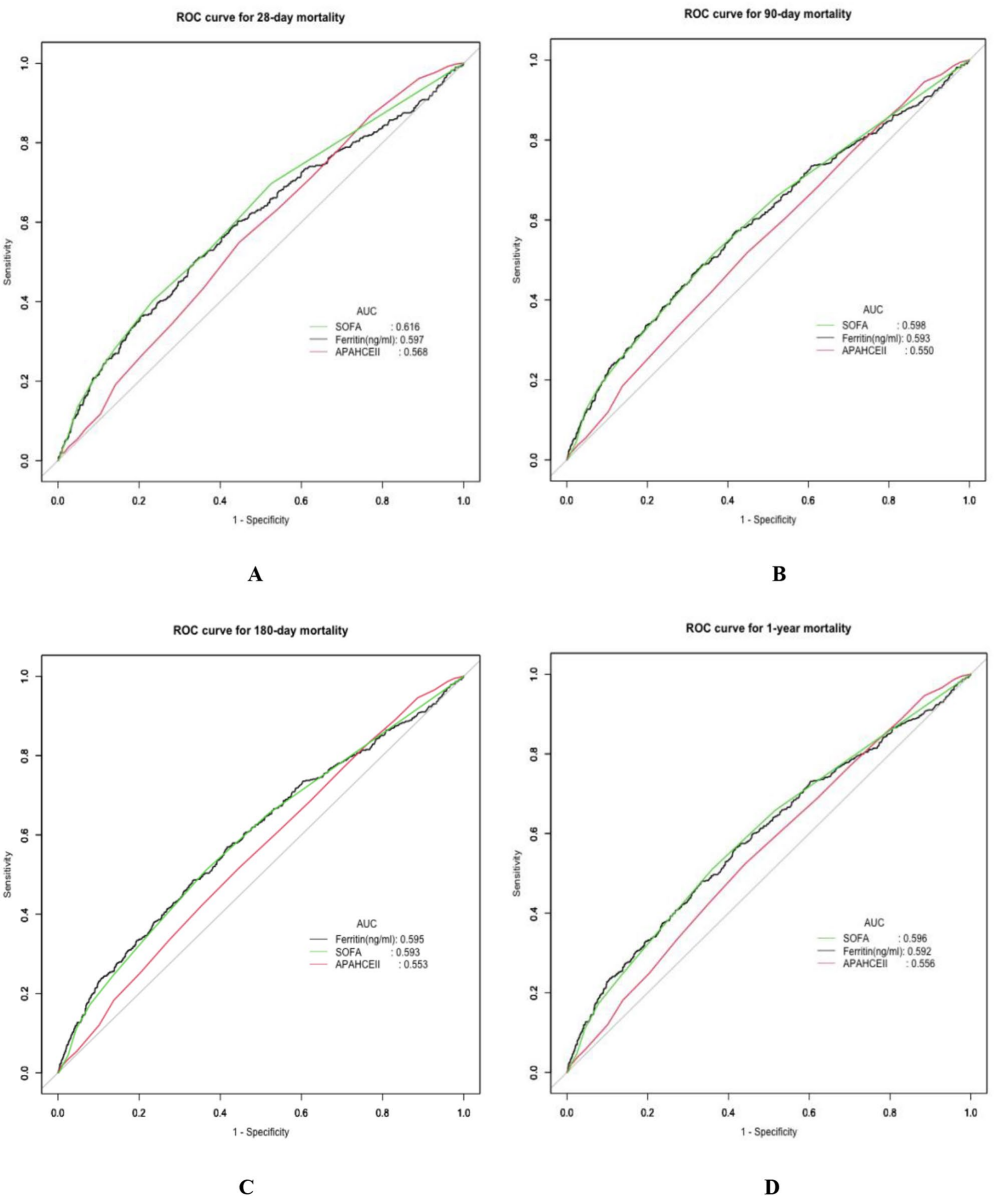
ROCs of ferritin, SOFA and APACHEII for predicting mortalities of 28-day (**A**), 90-day (**B**), 180-day (**C**) and 1-year (**D**) in sepsis. SOFA, sequential organ failure assessment; APACHE, acute physiology and chronic health evaluation.

In Table 5, predictive performances of serum ferritin, SOFA and APAHCEII in clinical outcomes were compared. The areas under the ROC curve (AUC) of ferritin for predicting mortalities of 28-day, 90-day, 180-day and 1-year were 0.597 (95% CI 0.563–0.629), 0.593 (95% CI 0.564–0.621), 0.595 (95% CI 0.567–0.623) and 0.592 (95% CI 0.564–0.620), respectively. The cut-off values of serum ferritin were 800.00 ng/ml, 599.50 ng/ml, 599.50 ng/ml and 597.5 ng/ml, respectively. In Fig. 3, ROCs of ferritin, SOFA and APACHEII for predicting mortalities of 28-day (A), 90-day (B), 180-day (C) and 1-year (D) in sepsis were demonstrated.

**Table 5:** Predictive performances of ferritin and scoring systems in clinical outcomes.

Variables	AUC	95% CI lower	95% CI upper	Cut-off value	Specificity	Sensitivity
28-day mortality						
Ferritin (ng/ml)	0.597	0.563	0.629	800.00	0.665	0.498
SOFA	0.616	0.584	0.646	3.00	0.474	0.697
APACHEII	0.568	0.537	0.598	13.00	0.552	0.549
*P*-value	0.002	–	–	–	–	–
90-day mortality						
Ferritin (ng/ml)	0.593	0.564	0.621	599.50	0.579	0.572
SOFA	0.598	0.570	0.624	4.00	0.634	0.516
APACHEII	0.550	0.522	0.578	13.00	0.552	0.518
*P*-value	< 0.001	–	–	–	–	–
180-day mortality						
Ferritin (ng/ml)	0.595	0.567	0.623	599.50	0.582	0.569
SOFA	0.593	0.566	0.619	4.00	0.636	0.510
APACHEII	0.553	0.525	0.580	13.00	0.553	0.516
*P*-value	0.001	–	–	–	–	–
1-year mortality						
Ferritin (ng/ml)	0.592	0.564	0.620	597.50	0.582	0.565
SOFA	0.596	0.570	0.622	4.00	0.639	0.511
APACHEII	0.556	0.528	0.582	13.00	0.558	0.524
*P*-value	0.003	–	–	–	–	–

AUC, area under the curve; CI, confidential interval; SOFA, sequential organ failure assessment; APACHE, acute physiology and chronic health evaluation; OR, odds ratio; CI, confidential interval.

## Discussion

In the present study, the non-linear relationships between serum ferritin and clinical outcomes in sepsis were found. For every 1000 ng/ml increment in serum ferritin, the risks in mortalities of in 28-day, 90-day, 180-day and 1-year increased by 13%, 15%, 16% and 17%, respectively. In addition, serum ferritin had a predictive value for outcomes in sepsis.

Ferritin, as a significant protein in iron metabolism, was involved both in the iron homeostasis and inflammatory process^23–25^. The potential prognostic and diagnostic values of serum ferritin level have been proved in various disorders^25–27^. In hemophgocytosis, a serum ferritin > 2000 ug/l for predicting mortality had a specificity of 76% and a sensitivity of 71%^28^. In hospitalized patients, ferritin levels greater than 2000 ng/ml were identified to be significantly associated with severe diseases^29^. In metabolic syndrome, the serum ferritin levels were found to be positively related with the levels of insulin resistance, cholesterol, and triglyceride^30^. One recent study with hemorrhagic fever with renal syndrome in China revealed that the value of serum ferritin for predicting mortality was comparatively good compared with procalcitonin and C-reactive protein^31^. For all-cause mortality risk within five-year in hemodialysis patients, serum ferritin > 1500 ng/ml was an early indicator^32^.

In critical ill and sepsis, accumulating evidences also clarified the close relationship between serum ferritin and clinical outcomes. In children with sepsis and septic shock, a ferritin > 500 ng/ml increased the relative risk of mortality with a 2.2 folds^33^. For predicting death in multiple organ dysfunction due to sepsis, 1994.3 ng/ml might be a cut-off value in serum ferritin^34^. Ferritin > 4420 ng/ml was described to be diagnostic of macrophage activation-like syndrome and predictive of short (10-day) and 28-day mortality in sepsis^35^. Current evidence suggested the biomarker of serum ferritin was good for immunotyping and providing immunomodulatory treatment in sepsis with encouraging results^36^. Based on the results from one large research in critical ill patients, the AUCs for ferritin in predicting in-hospital mortality and organ failure were 0.655 and 0.646, respectively. In sepsis, the AUCs for ferritin in predicting in-hospital mortality and organ failure were 0.628 and 0.608, respectively, which the cut-off values were 411 ng/ml and 581 ng/ml, respectively^37^, which were partly similar with our results.

The potential mechanisms why elevated serum ferritin levels were correlated with poorer outcomes in sepsis could be explained as follow: (1) The inflammation due to sepsis usually produces the endotoxin, which upregulates the ferritin coding gene and leads to increased levels of serum ferritin^38^; (2) Ferroptosis, as a way of cell death, might be mediated by different levels of serum ferritin, resulting in cellular injury and organ dysfunction^39^; (3) Inflammatory cytokines are able to upregulate the ferritin production, which in turn strengthens the release of proinflammatory and anti-inflammatory factors, leading to more severe inflammatory response and poor prognosis^40,41^.

The strength of our study was that we investigated the relationships between serum ferritin and clinical outcomes with short-term and long-term in sepsis and also explored the predictive values of serum ferritin. We found that serum ferritin was closely associated not only with short-term outcomes but also with 1-year mortality in sepsis patients. Moreover, serum ferritin also had a comparatively good value for predicting the outcomes in sepsis patients. It might help physicians to early differentiating the patients with higher risk of adverse outcomes.

Anyway, some limitations in the research were not avoided. First, due to the lack of some data, some other factors of iron metabolism including transferrin, free iron level, and ferritin saturation were not included. Second, due to our exclusion criteria, the proportion of excluded subjects with missing variables might cause bias in the relationships. Due to the lack of serum ferritin, a number of sepsis patients were excluded. Third, it was a retrospective study based on public database and the limitations of applicability for our results should be considered. In the study, we only used the U.S public database and didn’t validate our results in other cohorts. Further study with large samples in multiple centers and different regions should be done for validating our results.

## Conclusion

In the present study, the non-linear relationships between serum ferritin and clinical outcomes in sepsis were found. In addition, serum ferritin had a predictive value for outcomes in sepsis.

## Supplementary Information

Supplementary Figure 1.

Supplementary Information 2.

Supplementary Table 3.

Supplementary Table 4.

## Data Availability

The data that support the findings of this study are available from the Massachusetts Institute of Technology (MIT) and Beth Israel Deaconess Medical Center (BIDMC) but restrictions apply to the availability of these data, which were used under license for the current study, and so are not publicly available. Data are however available from the authors upon reasonable request and with permission of the Massachusetts Institute of Technology (MIT) and Beth Israel Deaconess Medical Center (BIDMC).
