# Supplementary figures and images for: The relationship between serum ferritin level and clinical outcomes in sepsis based on a large public database
Source: Sci Rep. 2023 May 29;13:8677. doi: 10.1038/s41598-023-35874-2 (PMC10225766; doi:10.1038/s41598-023-35874-2)

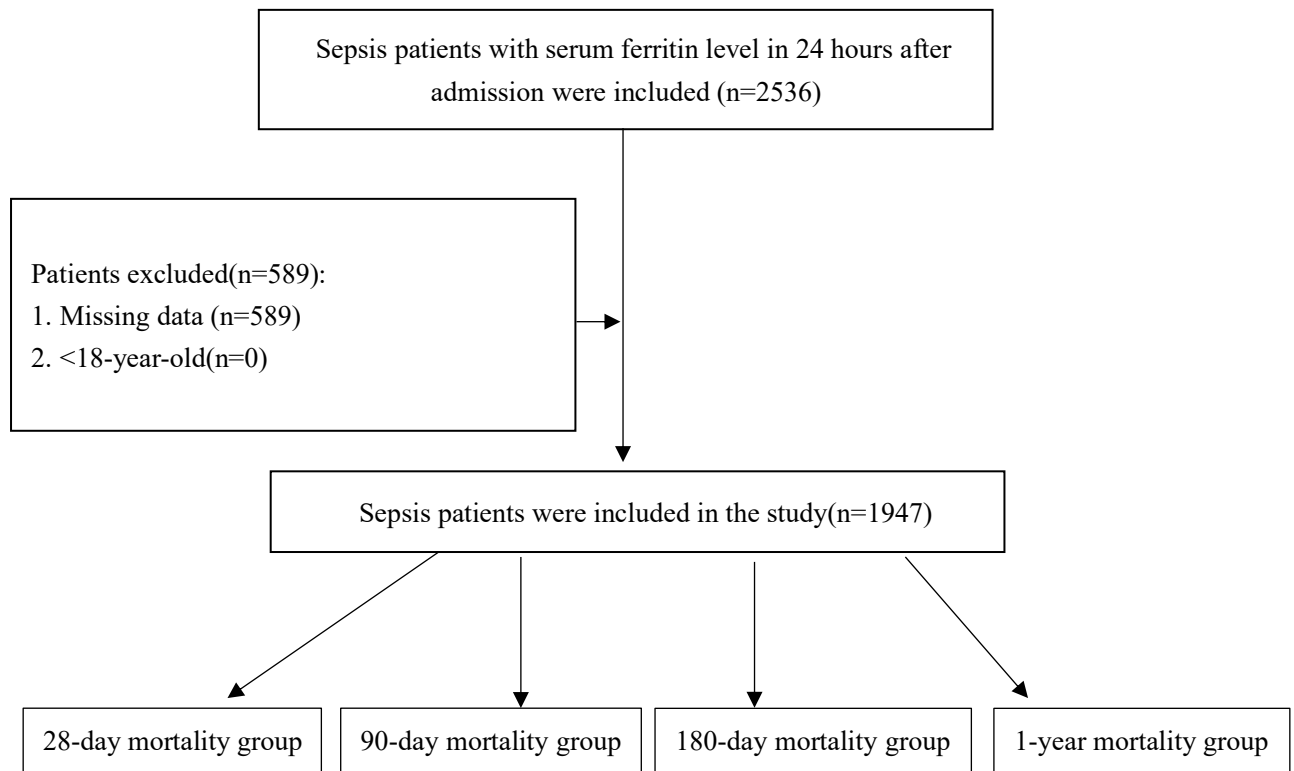

**Supplementary Figure 1: Flow chart and study design.**

Supplement: Supplementary file 1 — Supplementary Figure 1. [file 41598_2023_35874_MOESM1_ESM.pdf]
