# Supplementary material for: The relationship between serum ferritin level and clinical outcomes in sepsis based on a large public database
Source: Sci Rep. 2023 May 29;13:8677. doi: 10.1038/s41598-023-35874-2 (PMC10225766; doi:10.1038/s41598-023-35874-2)
Supplement: Supplementary file 2 — Supplementary Information 2. [file 41598_2023_35874_MOESM2_ESM.pdf]

Supplementary materials

Covariate checking and screening

First: VIF Collinearity screening

	Step 1	Step 2
Ferritin	1.2	1.2
Age	1.5	1.5
Gender	1.1	1.1
HR	1.2	1.2
DBP	1.6	1.6
SBP	1.6	1.6
RR	1.2	1.2
ALT	3.4	3.4
AG	8.5	2.1
AST	3.4	3.4
Bicarbonate	8.4	1.7
Total calcium	1.2	1.2
Chloride	16.3	NA
Creatinine	2.5	2.5
Hematocrit	9.2	9.2
Hemoglobin	8.4	8.4
PLT	1.4	1.4
PT	1.2	1.2
TT	1.1	1.1
RDW	1.3	1.3
RBC	4.7	4.7
Sodium	11.4	1.1
Urea nitrogen	2	2
WBC	1.2	1.2
Renal disease	1.2	1.2
CAD	1.1	1.1
Diabetes	1.1	1.1
Hypertension	1.2	1.1

APAHCEII	1.6	1.6
SOFA	2.3	2.3

Variables ruled out by collinearity screening：Chloride

Second: Checking the relationship between different covariates and 1-year mortality

Covariates	N	beta	Se.	exp(beta)	95%CI Low	95%CI Upp	P.value
Age	1947	0.0081	0.0032	1.0081	1.0018	1.0144	0.0119
Gender	1947	-0.0040	0.0981	0.9960	0.8217	1.2073	0.9676
HR	1947	0.0017	0.0022	1.0017	0.9974	1.0061	0.4307
DBP	1947	0.0001	0.0026	1.0001	0.9949	1.0053	0.9743
SBP	1947	-0.0014	0.0022	0.9986	0.9943	1.0029	0.5125
RR	1947	0.0052	0.0071	1.0052	0.9913	1.0193	0.4668
ALT	1947	0.0001	0.0001	1.0001	0.9999	1.0002	0.5037
AG	1947	0.0238	0.0092	1.0241	1.0059	1.0426	0.0094
AST	1947	0.0001	0.0001	1.0001	1.0000	1.0002	0.1323
Bicarbonate	1947	0.0152	0.0094	1.0153	0.9967	1.0343	0.1076
Total calcium	1947	0.1975	0.0500	1.2184	1.1047	1.3438	0.0001
Creatinine	1947	0.0212	0.0249	1.0215	0.9728	1.0725	0.3937
Hematocrit	1947	-0.0186	0.0077	0.9815	0.9669	0.9964	0.0150
Hemoglobin	1947	-0.0654	0.0233	0.9367	0.8948	0.9805	0.0050
PLT	1947	-0.0016	0.0004	0.9984	0.9977	0.9992	<0.0001
PT	1947	0.0090	0.0036	1.0091	1.0020	1.0162	0.0115
TT	1947	0.0110	0.0024	1.0111	1.0063	1.0159	<0.0001
RDW	1947	0.1607	0.0182	1.1743	1.1332	1.2169	<0.0001
RBC	1947	-0.2917	0.0662	0.7470	0.6560	0.8505	<0.0001
Sodium	1947	-0.0058	0.0077	0.9943	0.9794	1.0093	0.4520
Urea nitrogen	1947	0.0063	0.0016	1.0064	1.0031	1.0096	0.0001
WBC	1947	-0.0066	0.0055	0.9934	0.9829	1.0041	0.2260
Renal disease	1947	0.3615	0.2170	1.4355	0.9382	2.1964	0.0957
CAD	1947	0.1706	0.1734	1.1860	0.8442	1.6661	0.3254
Diabetes	1947	-0.0667	0.2790	0.9355	0.5415	1.6162	0.8110
Hypertension	1947	-0.0922	0.1298	0.9119	0.7070	1.1761	0.4775

APAHCEII	1947	0.0480	0.0114	1.0492	1.0260	1.0729	<0.0001
SOFA	1947	0.1703	0.0236	1.1857	1.1321	1.2419	<0.0001

Third: Introducing covariates into the basic model and removing covariates from the full model, checking the changes in the regression coefficient of X.

X= Ferritin(ng/ml)

	Basic model	Full model	
Covariates	Ferritin	Ferritin	Selection
	0.0001	0.0002	
Age	0.0002 *	0.0001 *	Yes
Gender	0.0001	0.0002	
HR	0.0001	0.0002	
DBP	0.0001	0.0002	
SBP	0.0001	0.0002	
RR	0.0001	0.0002	
ALT	0.0002 *	0.0001 *	Yes
AG	0.0001	0.0002	
AST	0.0001	0.0002	
Bicarbonate	0.0001	0.0002	
Total calcium	0.0002 *	0.0001 *	Yes
Creatinine	0.0001	0.0001 *	Yes
Hematocrit	0.0001	0.0001 *	Yes
Hemoglobin	0.0001	0.0002	
PLT	0.0001	0.0002	
PT	0.0001	0.0002	
TT	0.0001	0.0002	
RDW	0.0001	0.0002	
RBC	0.0001	0.0002	
Sodium	0.0001	0.0002	
Urea nitrogen	0.0001	0.0001 *	Yes
WBC	0.0001	0.0002	
Renal disease	0.0001	0.0002	
CAD	0.0001	0.0002	

Diabetes	0.0001	0.0002	
Hypertension	0.0001	0.0002	
APAHCEII	0.0001	0.0001 *	Yes
SOFA	0.0001	0.0002	

* Indicates a change of more than 10% compared to the initial regression coefficient.

Fourth: Covariates screening out by different standards

Y	X	Standard 1	Standard 2
1-year mortality	Ferritin(ng/ml)	Age, ALT, total calcium, creatinine, hematocrit, urea nitrogen, APAHCEII	Age; ALT; AG; total calcium; creatinine; hematocrit; hemoglobin; PLT; PT; TT; RDW; RBC; urea nitrogen; renal disease; APAHCEII; SOFA

Standard 1：The impact of introducing covariates into the basic model or removing covariates from the full model on the regression coefficient of X is greater than 10%
Standard 2： Standard 1 or The regression coefficient P value of the covariate to Y<0.1

Created by EmpowerStats (www.empowerstats.com) and R software
