# Supplementary material for: The relationship between serum ferritin level and clinical outcomes in sepsis based on a large public database
Source: Sci Rep. 2023 May 29;13:8677. doi: 10.1038/s41598-023-35874-2 (PMC10225766; doi:10.1038/s41598-023-35874-2)
Supplement: Supplementary file 3 — Supplementary Table 3. [file 41598_2023_35874_MOESM3_ESM.docx]

**Supplementary Table 1 General characteristics of sepsis patients**

| **Characteristics** |  |
| --- | --- |
| Number(median, IQR) | 1947 |
| Age(years） | 64.00 (53.00-74.00) |
| **Gender** (n,%) |  |
| Female | 867 (44.53%) |
| Male | 1080 (55.47%) |
| **Marital status**(n,%) |  |
| Married | 782 (40.16%) |
| Singled | 630 (32.36%) |
| Divorced | 154 (7.91%) |
| Others | 381(19.57%) |
| **Ethnicity**(n,%) |  |
| White | 1267 (65.07%) |
| Black/African American | 228 (11.71%) |
| Asian | 65 (3.34%) |
| Hispanic/Latino | 74 (3.80%) |
| Others | 313(16.08%) |
| **Managements**(n,%) |  |
| RRT | 445(22.85%) |
| Ventilator use | 1632(83.82%) |
| Vasopressor use | 516(26.50%) |
| **Organ dysfunction(**n,%**)**  AKI | 1475(75.76%) |
| Septic shock | 845(43.40%) |
| **Clinical outcomes** |  |
| 28-day mortality(n,%) | 393 (20.18%) |
| 90-day mortality(n,%) | 552 (28.35%) |
| 180-day mortality(n,%) | 590 (30.30%) |
| 1-year mortality(n,%) | 614 (31.54%) |

**Abbreviations:** RRT=renal replacement therapy, AKI=acute kidney injury.
