# Supplementary material for: The relationship between serum ferritin level and clinical outcomes in sepsis based on a large public database
Source: Sci Rep. 2023 May 29;13:8677. doi: 10.1038/s41598-023-35874-2 (PMC10225766; doi:10.1038/s41598-023-35874-2)
Supplement: Supplementary file 4 — Supplementary Table 4. [file 41598_2023_35874_MOESM4_ESM.docx]

**Supplementary Table 2: Subgroups and interaction analyses**

|  | N | **28-day mortality** | P | **90-day mortality** | P | **180-day mortality** | P | **1-year mortality** | P |
| --- | --- | --- | --- | --- | --- | --- | --- | --- | --- |
| Variables |  | OR(95%CI),P |  | OR(95%CI),P |  | OR(95%CI),P |  | OR(95%CI),P |  |
| Gender |  |  | 0.0115 |  | 0.0218 |  | 0.0176 |  | 0.0335 |
| Male | 1080 | 1.09 (1.03, 1.14) 0.0020 |  | 1.11 (1.05, 1.16) 0.0002 |  | 1.11 (1.05, 1.17) <0.0001 |  | 1.12 (1.06, 1.18) <0.0001 |  |
| Female | 867 | 1.22 (1.12, 1.33) <0.0001 |  | 1.24 (1.14, 1.36) <0.0001 |  | 1.26 (1.15, 1.38) <0.0001 |  | 1.25 (1.14, 1.37) <0.0001 |  |
| Age |  |  | 0.0099 |  | 0.0002 |  | <0.0001 |  | <0.0001 |
| ≤56 | 624 | 1.14 (1.07, 1.21) <0.0001 |  | 1.15 (1.08, 1.22) <0.0001 |  | 1.15 (1.08, 1.23) <0.0001 |  | 1.17 (1.09, 1.25) <0.0001 |  |
| 57-70 | 651 | 1.24 (1.13, 1.35) <0.0001 |  | 1.32 (1.19, 1.47) <0.0001 |  | 1.37 (1.22, 1.54) <0.0001 |  | 1.35 (1.20, 1.52) <0.0001 |  |
| ≥71 | 672 | 1.01 (0.91, 1.12) 0.8687 |  | 1.00 (0.90, 1.10) 0.9370 |  | 1.00 (0.91, 1.10) 0.9757 |  | 0.99 (0.90, 1.10) 0.9124 |  |
| Renal disease |  |  | 0.3733 |  | 0.8648 |  | 0.3439 |  | 0.4360 |
| No | 1853 | 1.13 (1.08, 1.18) <0.0001 |  | 1.14 (1.09, 1.19) <0.0001 |  | 1.14 (1.09, 1.20) <0.0001 |  | 1.15 (1.09, 1.20) <0.0001 |  |
| Yes | 94 | 1.04 (0.88, 1.24) 0.6200 |  | 1.16 (0.97, 1.38) 0.1038 |  | 1.27 (1.01, 1.60) 0.0451 |  | 1.25 (1.00, 1.57) 0.0530 |  |
| CAD |  |  | 0.1242 |  | 0.0354 |  | 0.0265 |  | 0.0194 |
| No | 1787 | 1.14 (1.09, 1.19) <0.0001 |  | 1.16 (1.10, 1.21) <0.0001 |  | 1.17 (1.11, 1.22) <0.0001 |  | 1.17 (1.12, 1.23) <0.0001 |  |
| Yes | 160 | 0.97 (0.77, 1.23) 0.8046 |  | 0.94 (0.76, 1.18) 0.6081 |  | 0.94 (0.75, 1.17) 0.5866 |  | 0.94 (0.75, 1.16) 0.5536 |  |
| Diabetes |  |  | 0.3177 |  | 0.1601 |  | 0.4447 |  | 0.3611 |
| No | 1884 | 1.13 (1.08, 1.18) <0.0001 |  | 1.15 (1.10, 1.20) <0.0001 |  | 1.15 (1.10, 1.21) <0.0001 |  | 1.16 (1.10, 1.22) <0.0001 |  |
| Yes | 63 | 0.95 (0.66, 1.39) 0.8075 |  | 0.92 (0.64, 1.32) 0.6513 |  | 1.04 (0.80, 1.36) 0.7730 |  | 1.02 (0.78, 1.34) 0.8594 |  |
| Hypertension |  |  | 0.9825 |  | 0.4736 |  | 0.2750 |  | 0.1509 |
| No | 1606 | 1.13 (1.07, 1.18) <0.0001 |  | 1.15 (1.09, 1.21) <0.0001 |  | 1.16 (1.10, 1.22) <0.0001 |  | 1.17 (1.11, 1.24) <0.0001 |  |
| Yes | 341 | 1.12 (1.01, 1.25) 0.0325 |  | 1.10 (0.99, 1.22) 0.0729 |  | 1.09 (0.98, 1.21) 0.1128 |  | 1.07 (0.97, 1.19) 0.1717 |  |
| HR |  |  | 0.1840 |  | 0.0690 |  | 0.0982 |  | 0.1083 |
| ≤87 | 636 | 1.08 (1.00, 1.17) 0.0504 |  | 1.09 (1.01, 1.17) 0.0266 |  | 1.10 (1.02, 1.19) 0.0124 |  | 1.10 (1.02, 1.19) 0.0152 |  |
| 88-108 | 630 | 1.11 (1.02, 1.20) 0.0111 |  | 1.11 (1.03, 1.20) 0.0061 |  | 1.12 (1.04, 1.21) 0.0044 |  | 1.14 (1.05, 1.23) 0.0017 |  |
| ≥109 | 681 | 1.19 (1.10, 1.28) <0.0001 |  | 1.24 (1.13, 1.35) <0.0001 |  | 1.24 (1.13, 1.36) <0.0001 |  | 1.24 (1.13, 1.37) <0.0001 |  |
| DBP |  |  | 0.2660 |  | 0.6586 |  | 0.5968 |  | 0.2600 |
| ≤56 | 633 | 1.19 (1.09, 1.29) <0.0001 |  | 1.18 (1.08, 1.29) 0.0002 |  | 1.19 (1.09, 1.30) 0.0002 |  | 1.23 (1.11, 1.36) <0.0001 |  |
| 57-70 | 653 | 1.10 (1.01, 1.19) 0.0275 |  | 1.12 (1.04, 1.22) 0.0042 |  | 1.15 (1.06, 1.24) 0.0011 |  | 1.14 (1.05, 1.23) 0.0021 |  |
| ≥71 | 661 | 1.10 (1.02, 1.18) 0.0112 |  | 1.13 (1.05, 1.21) 0.0012 |  | 1.12 (1.04, 1.21) 0.0021 |  | 1.12 (1.04, 1.20) 0.0028 |  |
| SBP |  |  | 0.0012 |  | 0.0027 |  | 0.0117 |  | 0.0210 |
| ≤102 | 639 | 1.08 (1.00, 1.17) 0.0386 |  | 1.10 (1.02, 1.19) 0.0139 |  | 1.11 (1.03, 1.20) 0.0092 |  | 1.13 (1.04, 1.23) 0.0031 |  |
| 103-122 | 651 | 1.31 (1.18, 1.46) <0.0001 |  | 1.34 (1.19, 1.51) <0.0001 |  | 1.32 (1.18, 1.49) <0.0001 |  | 1.31 (1.17, 1.47) <0.0001 |  |
| ≥123 | 657 | 1.06 (0.99, 1.14) 0.1054 |  | 1.09 (1.02, 1.16) 0.0134 |  | 1.10 (1.03, 1.18) 0.0040 |  | 1.10 (1.03, 1.18) 0.0052 |  |
| RR |  |  | 0.2666 |  | 0.7990 |  | 0.8005 |  | 0.9108 |
| ≤17 | 528 | 1.15 (1.05, 1.25) 0.0020 |  | 1.12 (1.02, 1.22) 0.0121 |  | 1.14 (1.04, 1.25) 0.0043 |  | 1.13 (1.04, 1.24) 0.0058 |  |
| 18-24 | 753 | 1.06 (0.98, 1.15) 0.1604 |  | 1.14 (1.05, 1.24) 0.0020 |  | 1.13 (1.04, 1.22) 0.0038 |  | 1.16 (1.06, 1.26) 0.0009 |  |
| ≥25 | 666 | 1.15 (1.08, 1.23) <0.0001 |  | 1.16 (1.08, 1.24) <0.0001 |  | 1.17 (1.09, 1.26) <0.0001 |  | 1.16 (1.08, 1.25) <0.0001 |  |
| ALT |  |  | 0.0242 |  | 0.1763 |  | 0.0422 |  | 0.0049 |
| ≤18 | 615 | 1.26 (1.11, 1.43) 0.0004 |  | 1.18 (1.05, 1.34) 0.0062 |  | 1.23 (1.08, 1.40) 0.0013 |  | 1.23 (1.08, 1.40) 0.0014 |  |
| 19-45 | 670 | 1.21 (1.08, 1.35) 0.0008 |  | 1.24 (1.10, 1.41) 0.0006 |  | 1.29 (1.12, 1.48) 0.0003 |  | 1.39 (1.19, 1.63) <0.0001 |  |
| ≥46 | 662 | 1.08 (1.02, 1.14) 0.0084 |  | 1.11 (1.05, 1.17) 0.0002 |  | 1.11 (1.05, 1.17) 0.0003 |  | 1.10 (1.04, 1.16) 0.0005 |  |
| AG |  |  | 0.3685 |  | 0.0756 |  | 0.0549 |  | 0.0316 |
| ≤13 | 544 | 1.13 (1.03, 1.25) 0.0102 |  | 1.13 (1.03, 1.24) 0.0113 |  | 1.16 (1.05, 1.27) 0.0037 |  | 1.20 (1.08, 1.33) 0.0007 |  |
| 14-18 | 736 | 1.17 (1.08, 1.28) 0.0003 |  | 1.24 (1.13, 1.37) <0.0001 |  | 1.25 (1.14, 1.38) <0.0001 |  | 1.26 (1.14, 1.39) <0.0001 |  |
| ≥19 | 667 | 1.09 (1.03, 1.16) 0.0042 |  | 1.09 (1.03, 1.16) 0.0029 |  | 1.09 (1.03, 1.16) 0.0030 |  | 1.09 (1.03, 1.16) 0.0050 |  |
| AST |  |  | 0.2090 |  | 0.5837 |  | 0.0007 |  | 0.0010 |
| ≤28 | 649 | 1.11 (0.96, 1.29) 0.1522 |  | 1.17 (1.02, 1.33) 0.0211 |  | 1.28 (1.11, 1.47) 0.0007 |  | 1.29 (1.12, 1.49) 0.0005 |  |
| 29-68 | 636 | 1.18 (1.08, 1.30) 0.0004 |  | 1.16 (1.06, 1.27) 0.0020 |  | 1.15 (1.05, 1.26) 0.0031 |  | 1.18 (1.07, 1.31) 0.0011 |  |
| ≥69 | 662 | 1.07 (1.02, 1.13) 0.0093 |  | 1.11 (1.05, 1.17) 0.0005 |  | 1.10 (1.04, 1.16) 0.0007 |  | 1.10 (1.04, 1.16) 0.0010 |  |
| Bicarbonate |  |  | 0.4222 |  | 0.4984 |  | 0.4007 |  | 0.3096 |
| ≤18 | 558 | 1.16 (1.08, 1.25) <0.0001 |  | 1.19 (1.10, 1.28) <0.0001 |  | 1.19 (1.10, 1.29) <0.0001 |  | 1.20 (1.11, 1.31) <0.0001 |  |
| 19-23 | 620 | 1.12 (1.03, 1.21) 0.0080 |  | 1.12 (1.03, 1.21) 0.0063 |  | 1.10 (1.02, 1.19) 0.0157 |  | 1.10 (1.02, 1.19) 0.0176 |  |
| ≥24 | 769 | 1.09 (1.00, 1.18) 0.0389 |  | 1.12 (1.04, 1.21) 0.0047 |  | 1.16 (1.06, 1.26) 0.0007 |  | 1.16 (1.07, 1.27) 0.0006 |  |
| Total calcium |  |  | 0.8267 |  | 0.2396 |  | 0.4518 |  | 0.3131 |
| ≤7.6 | 621 | 1.14 (1.07, 1.22) 0.0001 |  | 1.16 (1.08, 1.24) <0.0001 |  | 1.15 (1.08, 1.23) <0.0001 |  | 1.16 (1.08, 1.25) <0.0001 |  |
| 7.7-8.4 | 632 | 1.11 (1.02, 1.20) 0.0113 |  | 1.10 (1.02, 1.19) 0.0194 |  | 1.13 (1.04, 1.22) 0.0031 |  | 1.12 (1.03, 1.21) 0.0055 |  |
| ≥8.5 | 694 | 1.14 (1.05, 1.25) 0.0030 |  | 1.22 (1.10, 1.36) 0.0002 |  | 1.23 (1.10, 1.37) 0.0003 |  | 1.24 (1.11, 1.39) 0.0002 |  |
| Chloride |  |  | 0.5950 |  | 0.7731 |  | 0.6998 |  | 0.5287 |
| ≤99 | 640 | 1.09 (1.03, 1.16) 0.0050 |  | 1.12 (1.05, 1.19) 0.0009 |  | 1.12 (1.05, 1.20) 0.0008 |  | 1.12 (1.05, 1.19) 0.0011 |  |
| 100-106 | 553 | 1.13 (1.02, 1.25) 0.0153 |  | 1.16 (1.05, 1.28) 0.0042 |  | 1.17 (1.05, 1.29) 0.0029 |  | 1.17 (1.06, 1.30) 0.0024 |  |
| ≥107 | 754 | 1.15 (1.06, 1.24) 0.0004 |  | 1.15 (1.07, 1.25) 0.0003 |  | 1.16 (1.08, 1.26) 0.0002 |  | 1.18 (1.09, 1.28) <0.0001 |  |
| Creatinine |  |  | 0.2468 |  | 0.0369 |  | 0.0763 |  | 0.0039 |
| ≤0.9 | 553 | 1.19 (1.09, 1.30) 0.0002 |  | 1.27 (1.14, 1.41) <0.0001 |  | 1.26 (1.14, 1.40) <0.0001 |  | 1.35 (1.19, 1.53) <0.0001 |  |
| 1.0-2.1 | 711 | 1.11 (1.01, 1.23) 0.0249 |  | 1.09 (0.99, 1.19) 0.0764 |  | 1.12 (1.02, 1.22) 0.0198 |  | 1.11 (1.01, 1.22) 0.0274 |  |
| ≥2.2 | 683 | 1.09 (1.02, 1.15) 0.0058 |  | 1.10 (1.04, 1.17) 0.0013 |  | 1.11 (1.04, 1.18) 0.0011 |  | 1.10 (1.03, 1.17) 0.0025 |  |
| Hematocrit |  |  | 0.2182 |  | 0.2740 |  | 0.2150 |  | 0.0949 |
| ≤27.5 | 649 | 1.12 (1.04, 1.20) 0.0029 |  | 1.16 (1.07, 1.25) 0.0002 |  | 1.19 (1.09, 1.29) <0.0001 |  | 1.22 (1.12, 1.34) <0.0001 |  |
| 27.6-33.3 | 638 | 1.22 (1.10, 1.36) 0.0003 |  | 1.21 (1.09, 1.35) 0.0005 |  | 1.20 (1.08, 1.34) 0.0009 |  | 1.19 (1.07, 1.33) 0.0015 |  |
| ≥33.4 | 660 | 1.10 (1.03, 1.17) 0.0047 |  | 1.10 (1.03, 1.17) 0.0026 |  | 1.10 (1.03, 1.17) 0.0028 |  | 1.10 (1.03, 1.17) 0.0044 |  |
| Hemoglobin |  |  | 0.4515 |  | 0.2429 |  | 0.1029 |  | 0.0122 |
| ≤8.8 | 616 | 1.17 (1.07, 1.27) 0.0004 |  | 1.20 (1.10, 1.32) <0.0001 |  | 1.25 (1.13, 1.38) <0.0001 |  | 1.31 (1.17, 1.47) <0.0001 |  |
| 8.9-10.8 | 672 | 1.14 (1.05, 1.25) 0.0020 |  | 1.17 (1.07, 1.27) 0.0008 |  | 1.16 (1.06, 1.27) 0.0010 |  | 1.16 (1.06, 1.26) 0.0015 |  |
| ≥10.9 | 659 | 1.09 (1.03, 1.17) 0.0056 |  | 1.10 (1.03, 1.17) 0.0028 |  | 1.10 (1.03, 1.17) 0.0028 |  | 1.09 (1.03, 1.16) 0.0055 |  |
| PLT |  |  | 0.1617 |  | 0.9533 |  | 0.8871 |  | 0.7714 |
| ≤137 | 647 | 1.08 (1.02, 1.14) 0.0076 |  | 1.13 (1.07, 1.21) <0.0001 |  | 1.15 (1.08, 1.22) <0.0001 |  | 1.16 (1.08, 1.24) <0.0001 |  |
| 138-238 | 648 | 1.19 (1.06, 1.33) 0.0040 |  | 1.15 (1.02, 1.28) 0.0192 |  | 1.16 (1.03, 1.30) 0.0129 |  | 1.15 (1.03, 1.29) 0.0169 |  |
| ≥239 | 652 | 1.17 (1.07, 1.28) 0.0007 |  | 1.12 (1.03, 1.22) 0.0104 |  | 1.12 (1.03, 1.22) 0.0111 |  | 1.11 (1.02, 1.21) 0.0162 |  |
| PT |  |  | 0.1545 |  | 0.0765 |  | 0.0201 |  | 0.0203 |
| ≤13.8 | 647 | 1.21 (1.10, 1.34) 0.0001 |  | 1.25 (1.13, 1.39) <0.0001 |  | 1.30 (1.16, 1.46) <0.0001 |  | 1.31 (1.17, 1.47) <0.0001 |  |
| 13.9-17.8 | 646 | 1.11 (1.03, 1.20) 0.0080 |  | 1.09 (1.01, 1.17) 0.0326 |  | 1.08 (1.01, 1.17) 0.0321 |  | 1.10 (1.02, 1.19) 0.0128 |  |
| ≥17.9 | 654 | 1.09 (1.02, 1.16) 0.0106 |  | 1.12 (1.05, 1.20) 0.0007 |  | 1.12 (1.05, 1.20) 0.0007 |  | 1.12 (1.05, 1.20) 0.0012 |  |
| TT |  |  | 0.1538 |  | 0.3986 |  | 0.1834 |  | 0.2037 |
| ≤29.8 | 646 | 1.16 (1.03, 1.31) 0.0151 |  | 1.22 (1.08, 1.37) 0.0010 |  | 1.26 (1.12, 1.43) 0.0002 |  | 1.27 (1.12, 1.44) 0.0002 |  |
| 29.9-37.4 | 652 | 1.18 (1.09, 1.27) <0.0001 |  | 1.13 (1.05, 1.22) 0.0015 |  | 1.14 (1.05, 1.23) 0.0010 |  | 1.13 (1.05, 1.22) 0.0015 |  |
| ≥37.5 | 649 | 1.07 (1.01, 1.14) 0.0162 |  | 1.11 (1.05, 1.19) 0.0008 |  | 1.11 (1.04, 1.18) 0.0010 |  | 1.12 (1.05, 1.20) 0.0005 |  |
| RDW |  |  | 0.9311 |  | 0.6295 |  | 0.4725 |  | 0.2245 |
| ≤14.6 | 644 | 1.12 (1.03, 1.21) 0.0068 |  | 1.14 (1.06, 1.24) 0.0008 |  | 1.18 (1.09, 1.28) <0.0001 |  | 1.18 (1.09, 1.28) <0.0001 |  |
| 14.7-17.0 | 652 | 1.14 (1.06, 1.23) 0.0005 |  | 1.12 (1.04, 1.21) 0.0026 |  | 1.11 (1.03, 1.20) 0.0043 |  | 1.10 (1.03, 1.19) 0.0080 |  |
| ≥17.1 | 651 | 1.12 (1.04, 1.21) 0.0023 |  | 1.18 (1.09, 1.29) 0.0001 |  | 1.18 (1.08, 1.29) 0.0002 |  | 1.22 (1.11, 1.34) <0.0001 |  |
| RBC |  |  | 0.6525 |  | 0.4554 |  | 0.1683 |  | 0.0434 |
| ≤2.99 | 645 | 1.15 (1.06, 1.25) 0.0004 |  | 1.17 (1.08, 1.27) 0.0002 |  | 1.21 (1.11, 1.33) <0.0001 |  | 1.26 (1.14, 1.38) <0.0001 |  |
| 3.00-3.66 | 651 | 1.12 (1.04, 1.22) 0.0050 |  | 1.15 (1.06, 1.26) 0.0011 |  | 1.15 (1.06, 1.26) 0.0012 |  | 1.14 (1.05, 1.25) 0.0019 |  |
| ≥3.67 | 651 | 1.10 (1.02, 1.18) 0.0098 |  | 1.10 (1.03, 1.18) 0.0058 |  | 1.09 (1.02, 1.17) 0.0102 |  | 1.09 (1.02, 1.16) 0.0154 |  |
| Sodium |  |  | 0.8614 |  | 0.5185 |  | 0.6235 |  | 0.6526 |
| ≤134 | 566 | 1.14 (1.06, 1.23) 0.0009 |  | 1.18 (1.08, 1.28) 0.0002 |  | 1.18 (1.08, 1.29) 0.0002 |  | 1.18 (1.08, 1.29) 0.0002 |  |
| 135-140 | 724 | 1.11 (1.02, 1.20) 0.0099 |  | 1.10 (1.02, 1.19) 0.0107 |  | 1.12 (1.04, 1.21) 0.0040 |  | 1.12 (1.04, 1.21) 0.0037 |  |
| ≥141 | 657 | 1.12 (1.04, 1.21) 0.0019 |  | 1.15 (1.06, 1.24) 0.0004 |  | 1.15 (1.06, 1.24) 0.0005 |  | 1.17 (1.07, 1.26) 0.0002 |  |
| Urea nitrogen |  |  | 0.6937 |  | 0.3339 |  | 0.1539 |  | 0.0804 |
| ≤20 | 623 | 1.13 (1.05, 1.22) 0.0014 |  | 1.17 (1.08, 1.26) 0.0002 |  | 1.17 (1.08, 1.27) 0.0001 |  | 1.21 (1.10, 1.32) <0.0001 |  |
| 21-42 | 661 | 1.16 (1.05, 1.28) 0.0037 |  | 1.19 (1.08, 1.31) 0.0006 |  | 1.22 (1.10, 1.36) 0.0001 |  | 1.22 (1.10, 1.35) 0.0001 |  |
| ≥43 | 663 | 1.10 (1.04, 1.17) 0.0020 |  | 1.10 (1.03, 1.17) 0.0031 |  | 1.10 (1.03, 1.17) 0.0045 |  | 1.09 (1.02, 1.16) 0.0075 |  |
| WBC |  |  | 0.0435 |  | 0.0213 |  | 0.0366 |  | 0.0353 |
| ≤8.7 | 638 | 1.07 (1.00, 1.15) 0.0421 |  | 1.10 (1.03, 1.18) 0.0041 |  | 1.12 (1.04, 1.20) 0.0015 |  | 1.11 (1.03, 1.18) 0.0036 |  |
| 8.8-15.0 | 653 | 1.12 (1.05, 1.20) 0.0010 |  | 1.11 (1.04, 1.19) 0.0022 |  | 1.12 (1.04, 1.20) 0.0021 |  | 1.14 (1.06, 1.22) 0.0006 |  |
| ≥15.1 | 656 | 1.27 (1.13, 1.43) <0.0001 |  | 1.33 (1.17, 1.52) <0.0001 |  | 1.33 (1.16, 1.51) <0.0001 |  | 1.33 (1.16, 1.52) <0.0001 |  |
| SOFA |  |  | 0.0415 |  | 0.0909 |  | 0.0256 |  | 0.0156 |
| ≤2 | 856 | 1.25 (1.11, 1.40) 0.0001 |  | 1.23 (1.10, 1.38) 0.0003 |  | 1.28 (1.14, 1.44) <0.0001 |  | 1.27 (1.13, 1.43) <0.0001 |  |
| 3-4 | 570 | 1.09 (1.00, 1.20) 0.0492 |  | 1.13 (1.04, 1.24) 0.0066 |  | 1.14 (1.04, 1.25) 0.0053 |  | 1.18 (1.07, 1.31) 0.0010 |  |
| ≥5 | 521 | 1.06 (1.00, 1.12) 0.0389 |  | 1.08 (1.02, 1.14) 0.0108 |  | 1.08 (1.02, 1.14) 0.0116 |  | 1.07 (1.01, 1.13) 0.0203 |  |
| APACHEII |  |  | 0.9535 |  | 0.6269 |  | 0.4430 |  | 0.7405 |
| ≤9 | 539 | 1.13 (1.05, 1.21) 0.0017 |  | 1.14 (1.06, 1.23) 0.0005 |  | 1.13 (1.05, 1.22) 0.0011 |  | 1.15 (1.07, 1.24) 0.0003 |  |
| 10-14 | 678 | 1.12 (1.04, 1.21) 0.0018 |  | 1.12 (1.04, 1.20) 0.0026 |  | 1.13 (1.05, 1.22) 0.0013 |  | 1.14 (1.05, 1.23) 0.0010 |  |
| ≥15 | 730 | 1.14 (1.05, 1.24) 0.0014 |  | 1.18 (1.08, 1.29) 0.0002 |  | 1.21 (1.10, 1.33) <0.0001 |  | 1.19 (1.09, 1.30) 0.0002 |  |

**Abbreviations:** ALT=alanine aminotransferase, AST= aspartate aminotransferase, CAD= coronary artery disease, SBP=systolic blood pressure, DBP= diastolic blood pressure, HR= heart rate, RR=respiratory rate, WBC=white blood cells, PLT=platelet, RDW=red blood cell distribution width, RBC=red blood cells, PT= prothrombin time, TT=thrombin time, AG=anion gap, APACHE=acute physiology and chronic health evaluation, SOFA=sequential organ failure assessment.
